# Association between ossification of the longitudinal ligament of the cervical spine and arteriosclerosis in the carotid artery

**DOI:** 10.1038/s41598-020-60248-3

**Published:** 2020-02-25

**Authors:** Yasushi Oshima, Toru Doi, So Kato, Yuki Taniguchi, Yoshitaka Matsubayashi, Koji Nakajima, Fumihiko Oguchi, Hiroyuki Oka, Naoto Hayashi, Sakae Tanaka

**Affiliations:** 1 Department of Orthopaedic Surgery, The University of Tokyo, Tokyo, Japan; 2 Medical Research and Management for Musculoskeletal Pain, 22nd Century Medical & Research Center, The University of Tokyo, Tokyo, Japan; 3 Department of Computational Diagnostic Radiology and Preventive Medicine, The University of Tokyo, Tokyo, Japan

**Keywords:** Musculoskeletal system, Spinal cord diseases

## Abstract

Although several risk factors have been reported for cervical ossification of the longitudinal ligament (OPLL), most evaluations made in the past were based on plain X-ray, not on computed tomography (CT) scan. In this study, we aimed to clarify novel risk factors for cervical OPLL in asymptomatic subjects undergoing CT scan as their routine medical checkups. A total of 1789 Japanese asymptomatic subjects who underwent CT scan for the whole body as their routine medical checkups were retrospectively reviewed. The medical checkup also included laboratory examinations, bone mineral status, and ultrasound of the carotid artery. As a result, cervical OPLL was seen in 120 subjects (6.7%). As we compared the demographic and clinical data between subjects with and without OPLL, OPLL group showed older age, higher proportion of male sex, higher BMI, higher incidence of hypertension, higher levels of blood HbA1c and triglyceride, and higher incidence of plaques in the carotid artery. A multivariate logistic regression analysis revealed that age (Odds ratio (OR):1.03), male sex (OR: 1.91), and the presence of plaque in the carotid artery (OR: 1.71) were risk factors for OPLL. To the best of our knowledge, this is the first report to reveal an association between OPLL and arteriosclerotic lesions.

## Introduction

Ossification of the posterior longitudinal ligament (OPLL) is a disorder characterized by progressive ectopic OPLL. Although the precise mechanisms of OPLL remain uncertain, it is considered a multifactorial disease with both environmental and genetic factors; in addition, several risk factors have been reported, including age, male sex, ethnicity, presence of diabetes mellitus, obesity, diet, sleeping time, and mechanical stimulation^1–9^.

Most OPLL patients are diagnosed by X-ray or computed tomography (CT) scanning when they present with radiculopathy or myelopathy. When symptoms are severe with evidence of compression to the nerve root or spinal cord, surgical intervention is often required. However, past epidemiological studies revealed that the incidence of OPLL may be higher in the general population because some patients do not present with clinical symptoms regardless of the presence of OPLL^4,10^.

Most epidemiological studies of OPLL have used X-rays for diagnosis; importantly, CT scans can provide more accurate diagnoses of OPLL than X-rays. There has been one study comparing the incidence of OPLL among various ethnic groups by using CT scans^11^; however, there have been no previous reports regarding risk factors of OPLL based on diagnosis through CT scans.

In this study, we aimed to investigate novel risk factors for cervical OPLL in asymptomatic subjects undergoing their routine medical checkups, which included laboratory examinations, bone mineral status evaluation, carotid artery ultrasound, and whole-body CT scans. To the best of our knowledge, this is the first study to assess the association between OPLL and arteriosclerosis.

## Results

### Participant characteristics

Of the 1789 subjects enrolled, 1116 (62%) were men; the subjects’ mean age was 58.5 years (standard deviation = 11.2 years) and mean BMI was 23.7 kg/m^2^ (standard deviation = 3.5 kg/m^2^). The demographic data are shown in Table 1. Cervical OPLL was observed in 120 subjects (6.7%). The mean OPLL thickness was 3.7 mm (range, 2.0–8.4 mm), and the mean occupying ratio of OPLL to the spinal canal was 29.4% (range, 12%–62%). The OPLL types were as follow: continuous type in 16 subjects, segmental type in 91 subjects, mixed type in 7 subjects, and localized type in 6 subjects.

**Table 1:** Demographic data of subjects.

Total No of subjects	1789
Age	58.5 ± 11.2
Male Sex - no. (%)	1116 (62%)
BMI (kg/m^2^)	23.7 ± 3.5
Hypertension - no. (%)	303 (17%)
Current smoking - no. (%)	337 (19%)
HbA1c (cNGSP)	5.8 ± 0.7
TG (mg/dL)	117.8 ± 80.3
Total cholesterol (mg/dL)	200.5 ± 33.0
HDL cholesterol (mg/dL)	64.4 ± 17.3
LDL cholesterol (mg/dL)	121.7 ± 30.0
UA (mg/dL)	5.6 ± 1.4
Ca (mg/dL)	9.0 ± 0.3
P (mg/dL)	3.5 ± 0.4
Ultrasound of carotid artery	
IMT (cm)	0.84 ± 0.19
Presence of plaque - no. (%)	439 (25%)
Bone quantitative ultrasound	
BUA (dB/MHz)	109.9 ± 16.1
SOS (m/s)	1564.9 ± 48.5

BMI, body mass index; TG, triglyceride; HDL cholesterol, high-density lipoprotein cholesterol; LDL cholesterol, low-density lipoprotein cholesterol; UA, uric acid; IMT, intima-media thickness; BUA, broadband ultrasonic attenuation; SOS, speed of sound.

### Comparison between subjects with and without OPLL

Comparison of demographic and clinical data between subjects with and without OPLL showed that subjects in the OPLL group were older (63.3 vs. 58.2 years, p < 0.001), more likely to be men (80% vs. 61%, p < 0.001), and had higher BMI (24.8 vs. 23.6 kg/m^2^, p < 0.001) and higher incidence of hypertension (28% vs. 16%, p = 0.001) as well as higher levels of blood HbA1c (6.0% vs. 5.8%, p = 0.001), TG (132.6 vs. 116.7 mg/dL, p = 0.03), and UA (6.0 vs. 5.6 mg/dL, p = 0.001). Carotid artery ultrasound showed higher maximum IMT (0.88 vs. 0.84 cm, p = 0.02) and higher incidence of plaques (45% vs. 23%, p < 0.001) in subjects with OPLL. There were no significant differences in the other parameters. Dichotomization of continuous variables (BMI, HbA1c, TG, UA, and IMT) revealed significant differences in HbA1c and TG between subjects with and without OPLL (Table 2).

**Table 2:** Comparison between subjects with and without OPLL.

	OPLL	non OPLL	*p-value*
Total No of subjects	120	1669	
Age -yr	63.3 ± 10.4	58.2 ± 11.2	<0.001
Male Sex - no. (%)	96 (80%)	1020 (61%)	<0.001
BMI > 25 (kg/m^2^) - no. (%)	53 (44%)	513 (30%)	0.002
Hypertension - no. (%)	34 (28%)	269 (16%)	0.001
HbA1c > 6.5% - no. (%)	24 (20%)	185 (11%)	0.003
TG > 150 mg/dL- no. (%)	35 (29%)	348 (21%)	0.03
UA > 7.0 mg/dL - no. (%)	25 (21%)	278 (17%)	0.239
IMT > 1.1 cm - no. (%)	20 (17%)	224 (13%)	0.33
Presence of plaque - no. (%)	54 (45%)	385 (23%)	<0.001

OPLL, ossification of the posterior longitudinal ligament; BMI, body mass index; HbA1c, glycated hemoglobin; TG, triglyceride; UA, uric acid; IMT, intima-media thickness.

### Risk factors for cervical OPLL

Finally, multivariate logistic regression analysis revealed that older age, male sex, and the presence of plaque in the carotid artery were risk factors for OPLL (Table 3).

**Table 3:** Multivariate logistic regression analysis as risk factors for OPLL.

	OR	95%CI			p value
Age	1.03	1.01	—	1.05	0.00
Male Sex	1.91	1.18	—	3.09	0.01
BMI (>25)	1.34	0.90	—	2.00	0.15
Hypertension	1.54	1.00		2.37	0.05
HbA1c (>6.5)	1.23	0.75	—	2.02	0.41
TG (>150)	1.37	0.88	—	2.13	0.16
Presence of plaque	1.71	1.13	—	2.58	0.01

OPLL, ossification of the posterior longitudinal ligament; BMI, body mass index; HbA1c, glycated hemoglobin; TG, triglyceride; OR, odds ratio; CI, confidence interval.

## Discussion

We identified several risk factors including older age, male sex, higher BMI, histories of diabetes mellitus and hyperlipidemia, and the presence of plaques in the carotid artery. In particular, older age, male sex, and plaque in the carotid artery remained significant risk factors after adjustment in multivariate analysis. As far as we know, this is the first report to reveal an association between OPLL and arteriosclerotic lesions.

OPLL was previously considered specific to the Asian population, particularly Japanese individuals, with an incidence of 1.9%–4.3% in the general population in Japan^10^. The incidences of OPLL in outpatient clinics were 0.4%–3.0% in Asian countries other than Japan and 0.1–1.7% in Europe and the United States^10^; however, there have been no studies regarding the incidence of OPLL in the general population. Therefore, the incidence of OPLL is considered to be higher in Japan, as well as in other Asian countries, compared with that in Western countries. Thus, ethnicity is considered to be related to the onset of OPLL.

The mechanism underlying the onset of OPLL remains uncertain, although it is presumably related to cytokine activity. For example, insulin-like growth factor I, which is reportedly involved in osteogenesis, was shown to be dominantly expressed in ligament cells derived from ossified spinal ligaments^12^. Bone morphogenetic protein-2 and transforming growth factor-β are also reportedly related to the pathogenesis of OPLL^13^. Similarly, inflammatory cytokines, IL-6 and TNF-α, have been associated with the process of yellow ligament ossification^14^. Moreover, involvement of the Wnt/β-catenin signaling pathway has been suggested in patients with OPLL^15^. Recently, a genome-wide association study of OPLL patients identified *RSPO2*, which encodes R-spondin 2, as a susceptibility gene for OPLL; notably, R-spondin 2 is a secreted agonist of canonical Wnt–β-catenin signaling and is reduced in early stages of chondrocyte differentiation^16^. In addition, Kawaguchi *et al*. reported that serum high-sensitivity C-reactive protein was elevated in OPLL patients, compared with that in individuals without OPLL; this indicated that local inflammation was associated with the progression of OPLL^17^. In a related manner, the level of serum C-reactive protein was high in patients with heterotopic ossification who underwent total hip arthroplasty^18^. Taken together, these prior findings support the involvement of inflammatory cytokines in the pathogenesis of OPLL, although the precise mechanisms remain uncertain.

In this study, arteriosclerosis was observed in the carotid artery more frequently in subjects with OPLL; this condition is reportedly a high-risk factor for stroke and is associated with atherosclerotic lesions in the coronary artery^19,20^ It is well known that inflammatory cytokines play important roles in the pathogenesis of arteriosclerosis^21^. For example, TNF-α and IL-6 were significantly associated with the prevalences of clinical and subclinical disease, as well as the incidence of cardiovascular events^22^. Aging, which was identified as an independent risk factor for OPLL in the present study, has also been associated with increased levels of circulating inflammatory cytokines, including IL-6 and TNF-α^23^. Given that inflammatory cytokines are associated with ossification of the paraspinal ligaments, as well as with aging and arteriosclerosis, we speculate that this is a common factor that led to the associations of aging and plaques in the carotid artery with risk of OPLL in the present study. Further comparative studies that include patients with massive OPLL are necessary to confirm this hypothesis.

The incidence of OPLL in Japan was 6.7% in this study, which was comparable with the incidence reported in a previous study of subjects undergoing CT scans for cancer screening^11^. Because our study also utilized data from medical screenings for cancer, the incidence of OPLL may be different from that in the general population. Nevertheless, we consider it to be reasonable that the incidence in our study was higher than that in previous reports involving diagnosis by plain X-rays, as CT scans can detect smaller areas of OPLL than X-rays. Thus, the majority of subjects with OPLL in our study was not considered to have pathologic disease and is not expected to present with severe myelopathy in the future. Indeed, the average occupying ratio of OPLL to the spinal canal was 29% in this study. On the basis of a nationwide survey in Japan, the maximum spinal canal stenosis rates were 38% and 27% in patients with and without myelopathy, respectively^10^. Matsunaga *et al*. prospectively investigated 450 patients with cervical OPLL over a mean follow-up period of 17.6 years (10–30 years); 323 of these patients did not exhibit myelopathy. Kaplan–Meier analysis showed that the myelopathy-free survival rate was 71% at 30 years^24^. Hence, we speculate that most subjects with OPLL in the present study may differ from myelopathic patients with massive OPLL and will not develop myelopathy. However, we acknowledge that the pathogenesis of ligament ossification is similar regardless of the extent of OPLL and that myelopathic symptoms result from the dynamic factor of the neck spine, as well as the extent of OPLL. Nevertheless, further studies involving myelopathic patients are needed to confirm the possibility of an interaction between OPLL and arteriosclerosis.

There were several limitations in this study. First, subject selection might have been biased because we recruited subjects undergoing voluntary medical checkups. From medical interviews, we confirmed that subjects were generally asymptomatic and did not have severe spinal disorders. Therefore, the incidence of OPLL may not be generalizable to the broader population. Second, patients with large areas of OPLL were not included in this study. As stated earlier in this section, smaller areas of OPLL rarely progress into large areas that require surgical intervention. Thus, most cases of OPLL in this study might differ from cases of OPLL that result in severe myelopathy. Third, we did not have detailed information on carotid plaques, such as the size and echo intensity. Because carotid plaques are pathologically classified into several types, such as necrotic core, intraplaque hemorrhage, fibrous components, and calcification, OPLL may be associated with calcification in carotid plaque, not with atherosclerosis. Finally, there may be other confounding factors that could not be adjusted, although we performed multivariate regression analysis. Further studies are needed to address these issues.

In conclusion, age, male sex, and the presence of plaques in the carotid artery were identified as risk factors for OPLL. This is the first report to reveal an association between OPLL and arteriosclerotic lesions, although the subjects enrolled in this study might be biased.

## Materials and Methods

This retrospective study was approved by the Institutional Review Board at the authors’ institution (No. 2674–2). The study was performed in accordance with approved guidelines and in compliance with the principles of the Declaration of Helsinki. All participants provided written informed consent before undergoing any study procedure.

A total of 1789 asymptomatic subjects who underwent whole-body CT scans during routine medical checkups from January 2011 to December 2011 were prospectively enrolled and included in this study. Medical checkups also included laboratory examinations, bone mineral status evaluation, and carotid artery ultrasound. We compared the results of all assessments between subjects with and without OPLL. Furthermore, multivariate logistic regression analysis was performed to identify the risk of OPLL.

All CT volumes were scanned by using a GE Light Speed Scanner (GE Healthcare Japan, Tokyo, Japan; 120 kV, 80 mA, 512 × 512 matrix), for the whole body ranging from the head to the proximal femur. Axial CT images were observed in the bone window with automatic adjustment. OPLL was defined as ossification of the posterior longitudinal ligament with >2 mm thickness in the axial view of the cervical spine^11,25^ The occupying ratio was also measured, comprising the maximum anterior-to-posterior ratio of the OPLL to the spinal canal in the axial slice.

Bone mineral status was examined by quantitative ultrasound using the A-1000 EXP II (GE Healthcare Japan, Tokyo, Japan), which has been broadly utilized for the assessment of bone mineral density at medical checkups. The parameters included broadband ultrasound attenuation, which measures the frequency dependence of ultrasound attenuation, and speed of sound, which reflects the transmission velocity of ultrasound passing through soft tissue.

Regarding ultrasound examination of the carotid artery, the maximum intima-media thickness (IMT) in the common carotid artery was defined as the maximum measurable IMT in scanned common carotid artery areas^19^. The presence of plaque was judged by trained readers using the presence or absence of two of the following three criteria: abnormal wall thickness (carotid IMT > 1.1 mm), abnormal shape (protrusion into the lumen and loss of alignment with adjacent arterial wall boundary), and abnormal wall texture (brighter echoes than adjacent boundaries)^26^. The Aplio 300 (Canon Medical Systems Corporation, Tochigi, Japan) was used for all ultrasound examinations.

### Statistical analysis

Continuous variables were compared using Student’s t-test, and categorical variables were compared using the chi-squared test. Continuous variables in laboratory examinations of blood samples were categorized in terms of normal upper limit and the need for treatment (glycated hemoglobin [HbA1c], triglyceride [TG], and uric acid [UA]). Body mass index (BMI) was categorized with a cutoff value of 25.0 kg/m^2^, as the average in this study was 23.7 kg/m^2^, and a BMI of 30.0 kg/m^2^ is too high for the Japanese population, although a BMI of 30.0 is often used as a cutoff value for obesity. Hypertension was defined as a systolic blood pressure of 140 mm Hg or more, and/or a diastolic blood pressure of 90 mm Hg or more. Multivariate logistic regression analysis was used to compute odds ratios and 95% confidence intervals to identify risk factors associated with OPLL. IBM SPSS Statistics software version 22.0 (IBM Corporation, Armonk, NY, USA) was used for all analyzes. All p values were two sided, and p values < 0.05 were considered statistically significant.
